# Automated speech artefact removal from MEG data utilizing facial gestures and mutual information

**DOI:** 10.1162/imag_a_00545

**Published:** 2025-04-22

**Authors:** Sara Tuomaala, Salla Autti, Silvia Federica Cotroneo, Pantelis Lioumis, Hanna Renvall, Mia Liljeström

**Affiliations:** Department of Neuroscience and Biomedical Engineering, Aalto University School of Science, Espoo, Finland; BioMag Laboratory, HUS Medical Imaging Center, Helsinki University Hospital, Helsinki University and Aalto University School of Science, HUS Helsinki, Finland; Aalto NeuroImaging, Aalto University, 00076 Aalto, Finland

**Keywords:** speech artefact removal, independent component analysis, magnetoencephalography, mutual information

## Abstract

The ability to speak is one of the most crucial human skills, motivating neuroscientific studies of speech production and speech-related neural dynamics. Increased knowledge in this area allows, for example, for development of rehabilitation protocols for language-related disorders. While our understanding of speech-related neural processes has been greatly enhanced owing to non-invasive neuroimaging techniques, the interpretations have been limited by speech artefacts caused by the activation of facial muscles that mask important language-related information. Despite earlier approaches applying independent component analysis (ICA), the artefact removal process continues to be time consuming, poorly replicable, and affected by inconsistencies between different observers, typically requiring manual selection of artefactual components. The artefact component selection criteria have been variable, leading to non-standardized speech artefact removal processes. To address these issues, we propose here a pipeline for automated speech artefact removal from magnetoencephalography (MEG) data. We developed an ICA-based speech artefact removal routine by utilizing electromyography (EMG) data measured from facial muscles during a facial gesture task for isolating the speech-induced artefacts. Additionally, we used mutual information (MI) as a similarity measure between the EMG signals and the ICA-decomposed MEG to provide a feasible way to identify the artefactual components. Our approach efficiently and in an automated manner removed speech artefacts from MEG data. The method can be feasibly applied to improve the understanding of speech-related cortical dynamics, while transparently evaluating the removed and preserved MEG activation.

## 1 Introduction

Communication through speech is crucial for humans, and an important part of our well-being. Over the last decades, our understanding of the neural dynamics underlying speech production has greatly improved owing to non-invasive studies utilizing electrophysiological techniques such as magnetoencephalography (MEG) and electroencephalography (EEG). Increased knowledge of the cortical functions related to speech and language allows, for example, for the development of rehabilitation protocols for language-related disorders, and increased precision in presurgical mapping of eloquent speech areas before resection in cases of tumour and drug-resistant epilepsy. However, in basic research and clinical settings utilizing MEG or EEG, identifying brain regions vital for speech is hampered by the prominent artefacts produced during speech.

Artefacts during vocal speech originate from various sources, such as the facial muscles and the movements of the head and the jaw (Aristei et al., 2011;Fargier et al., 2018;Ouyang et al., 2016). In addition, the tongue movement produces a prominent artefact, the glossokinetic potential (Vanhatalo et al., 2003). The largest facial muscles activated during speech are the masseter and zygomaticus major, but most facial muscles used for speech are small and largely overlapping in their activity (Stepp, 2012). As the muscle artefacts arise from many independent muscle groups, it is difficult to characterize them and thus challenging to remove them from the MEG and EEG signals (McMenamin et al., 2010;Urigüen & Garcia-Zapirain, 2015). Moreover, muscular activity, as measured with surface electromyography (sEMG), has a wide spectral range from almost 0 to >200 Hz, typically peaking around 100 Hz (Urigüen & Garcia-Zapirain, 2015). The spectral contents of the recorded electrophysiological signals from the brain thus overlap with the EMG activity (Jiang et al., 2019;McMenamin et al., 2010;Urigüen & Garcia-Zapirain, 2015), further complicating the artefact removal.

Picture naming offers a convenient way to study the neural underpinnings of word production (Indefrey & Levelt, 2004;Levelt et al., 1998;Salmelin et al., 1994), since it involves the natural sequence of language production from conceptual processing to word articulation. It is also the most common task used to evaluate speech errors induced by brain stimulation in presurgical evaluation and during awake craniotomy (Corina et al., 2010;Krieg et al., 2017). Even though picture naming and its variations are widely used in both experimental brain research and clinical settings (Kohn & Goodglass, 1985;Krieg et al., 2017;Lioumis et al., 2012;Raffa et al., 2022;Sonoda et al., 2022), the prominent muscle artefacts limit the usage of vocal speech tasks during MEG and EEG recordings. Many attempts have been made to overcome this problem by modifying the task to avoid oral responses. For example, delayed (Ala-Salomäki et al., 2021;Jescheniak et al., 2002), silent (Eulitz et al., 2000;Liljeström et al., 2009), whispering (Eulitz et al., 2000), and manual responses (Schmitt et al., 2000) have been used. In addition, some studies have limited the data analysis to the time window before the onset of the speech response, when muscular artefacts do not yet contaminate the measured brain activation (Habets et al., 2008;Liljeström et al., 2015). Yet, despite the prominent muscular artefacts, using a vocal naming response has remarkable experimental advantages as it tracks the full word production process and ensures that one can assess whether the subject follows the given test instructions and names the pictures correctly. The need to remove the muscular contamination from the brain signals in vocal naming thus prevails.

Different methods for speech artefact removal have been proposed, such as removing the high-frequency muscular signal from the recordings by a low-pass or band-pass filtering (Ganushchak & Schiller, 2008;Laganaro & Perret, 2011), or applying blind source separation by canonical correlation analysis (Vos et al., 2010) to identify and remove the artefactual components. Additionally, algorithms utilizing independent component analysis (ICA), with different component selection criteria, have been previously suggested. Such criteria have included, for example, statistical characteristics such as kurtosis (Barbati et al., 2004;Porcaro et al., 2015), correlation of the components with the concurrent EMG signals (Alexandrou et al., 2017;Porcaro et al., 2015;Vos et al., 2010), or their power spectrum densities (PSDs) (Barbati et al., 2004;Porcaro et al., 2015), visual inspection of the component topography (Alexandrou et al., 2017;Hipp et al., 2011;Kujala et al., 2013;Porcaro et al., 2015), component localization at the source level (Alexandrou et al., 2017;Henderson et al., 2013;Porcaro et al., 2015), and the relative amount of gamma (∼40Hz)/high-gamma (>80Hz) activation in their spectral power (Alexandrou et al., 2017;Hipp et al., 2011). Despite the proposed methods, muscle artefact removal from speech data remains challenging and time consuming, and the cleaning process still relies on manual work. With manual component selection criteria, the artefact removal process may be poorly replicable and hampered by problems in inter-observer reliability (Gross et al., 2013;Muthukumaraswamy, 2013). Conversely, the more automatized selection criteria tend to vary between studies leading to non-standardized speech artefact removal processes. Evidently, there is a need for a reproducible and automatized speech artefact removal pipeline, with rather simple component selection criteria.

This study aims to improve the artefact removal process and proposes a replicable pipeline for automated speech artefact removal from MEG data. We designed a facial gesture task to be performed before the actual naming task for collecting examples of the most prominent facial muscular artefacts. The facial gesture task contains 10 movements that use the muscles essential to speech; muscle activity is measured over four facial muscles with EMG. Component selection is automated using mutual information (MI) between the EMG signals and the ICA-decomposed MEG signals. Even though correlation has been previously used in the context of identifying, for example, ocular artefacts from M/EEG recordings, mutual information has conceptual advantages as a similarity measure. For instance, correlation can be estimated to be zero when comparing two highly dependent non-Gaussian datasets, whereas, in the same example, mutual information can still correctly quantify the dependence (Hudson, 2006). The use of MI over correlation as a similarity measure is, therefore, motivated by its ability to find non-linear dependencies between pairs of signals. Moreover, we suggest that its better resilience to outliers compared with correlation (Correa & Lindstrom, 2013) makes it a more suitable tool for analyzing signals that contain high-frequency noise, such as the one originating from muscle activity. Similar approaches have shown promise for extracting deep brain stimulation (DBS) artefacts from MEG data (Abbasi et al., 2016) and cardio-ballistic artefacts from EEG signals (Abbasi et al., 2015;Liu et al., 2012). As a similarity measure, MI requires setting a threshold to define when two signals are considered similar enough. To solve this issue, we employed k-means clustering. We compared two different approaches: mutual information (MI) with clustering (C), denoted as MIC-ICA, and manual classifying of the artefactual components (Manual-ICA).

We evaluated whether the addition of a facial gesture task and the use of MI as a similarity measure can improve the artefact removal process in healthy subjects performing a picture naming task. The results were assessed from two perspectives: firstly, by evaluating how well the artefact removal process preserves true brain activation, and secondly, by measuring the success of the automated artefact removal process. Preservation of real brain activation was evaluated by comparing the cleaned data in a vocal naming task with that collected during silent naming, and the evaluation of the automation process was based on a comparison between a current standard Manual-ICA and the proposed MIC-ICA approach. Our results suggest that using the facial gesture task and simultaneous EMG measurements enables effective automation of the speech artefact reduction process in language-related MEG tasks with vocal speech responses, such as picture naming.

## 2 Materials and Methods

**Fig. 1.**
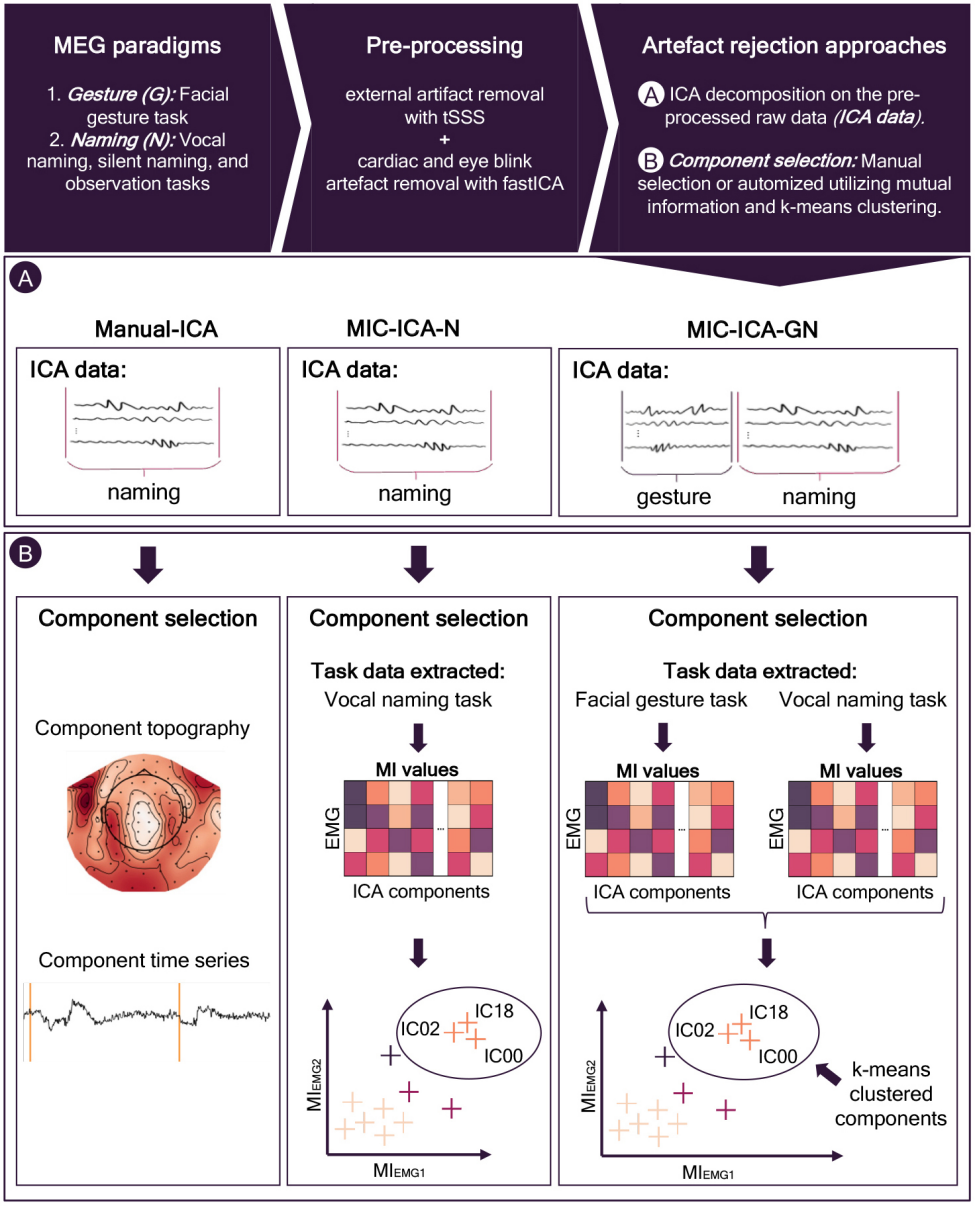
The study pipeline. The study consisted of a facial gesture paradigm (G) and a standard picture naming paradigm (N), consisting of vocal naming, silent naming, and observation tasks. The MEG data were accompanied by EMG, EOG, and ECG recordings. The measured data were pre-processed using tSSS and FastICA to remove external noise, eye blinks, and heart artefacts. To remove speech artefacts, FastICA was applied again, now to the pre-processed data: Manual-ICA and MIC-ICA-N used the data from the naming paradigm, and MIC-ICA-G additionally included the data from the gesture paradigm. In the Manual-ICA approach, the artefact ICA components were selected manually. In the MIC-ICA methods, mutual information values between the EMG signals and ICA-decomposed MEG signals were calculated. Components sharing high MI values were then identified with k-means clustering and subsequently removed.

Figure 1illustrates the overall study paradigm and the analysis workflow. First, all participants took part in a facial gesture paradigm, followed by a standard picture naming paradigm, which included vocal and silent naming tasks and a picture observation task. Hereafter, the picture naming paradigm refers to all these three subexperiments together (vocal naming, silent naming, and observation), while vocal and silent naming tasks refer specifically to the individual tasks. Electrocardiogram (ECG) and electrooculogram (EOG) were recorded simultaneously with the EMG and MEG data. The data were first pre-processed (see below for details) such that prominent external noise originating outside the helmet, eye blinks, and heart artefacts was removed, while the speech artefact remained. The differences between the three speech artefact rejection approaches were then analyzed. These approaches included two automated rejection methods that utilized mutual information (MI) and clustering (C) (MIC-ICA) to select the artefact components. In MIC-ICA-N, the ICA decomposition was applied to the naming paradigm data alone, while in MIC-ICA-GN, the ICA decomposition was applied to both the gesture and naming paradigm data. In the MIC-ICA approaches, component selection used mutual information (MI) between the measured facial EMGs and independent components as the metric for k-means clustering to automatically classify the components to be removed. For comparison, as a reference method, the artefactual components were classified manually based on visual inspection of their time series and topographies (Manual-ICA).

### 2.1 Subjects

In total, 12 healthy volunteers [mean ± STD age 25 ± 3 years, range 21–29, 6 females] participated in the study. All subjects were right handed, without neurological or psychiatric disorders and had Finnish as their native language. The measurement protocol was approved by the Helsinki University Hospital (HUS) Regional Committee on Medical Research Ethics, and all participants signed a written consent form to take part in the study.

### 2.2 Experimental design

#### 2.2.1 Facial gesture task

Before the picture naming paradigm, the participants performed a facial gesture task designed to aid in the automated identification of artefacts. Ten facial movements were performed, each designed to preferentially activate a specific facial muscle. These movements covered gestures that are known to produce strong but non-isolated muscular activations (O’Dwyer et al., 1981;Stepp, 2012; seeTable 1).

**Table 1.** The major muscles activated during speech and the gestures in which they are preferentially used.

	Muscle	Gesture
G1	Levator labii superioris	Unilateral snarl elevating the upper lip
G2	Zygomaticus major	Broad smile
G3	Buccinator	Puffing out the cheeks with the lips closed
G4	Risorius	Broad smile with the lips closed
G5	Orbicularis oris superioris	Compressing the upper lip against the upper incisors
G6	Orbicularis oris inferioris	Compressing the lower lip against the lower incisors
G7	Depressor labii inferioris	Pulling down the lower lip with the jaw closed
G8	Depressor anguli oris	Pulling down the corners of the mouth
G9	Mentalis	Raising and everting the lower lip while wrinkling the chin
G10	Genioglossus	Press the tongue against the palate

O’Dwyer et al. (1981).

A block diagram of the facial gesture task is presented inFigure 2. The overall task (Level 1) consisted of five sets of equal length (160 s). Within each set (Level 2), each of the 10 gestures, listed inTable 1, was performed in a randomized order. Each of the gestures within a set was repeated 5 times (Level 3). The subjects were prompted with an audio stimulus to perform the gesture.

**Fig. 2.**
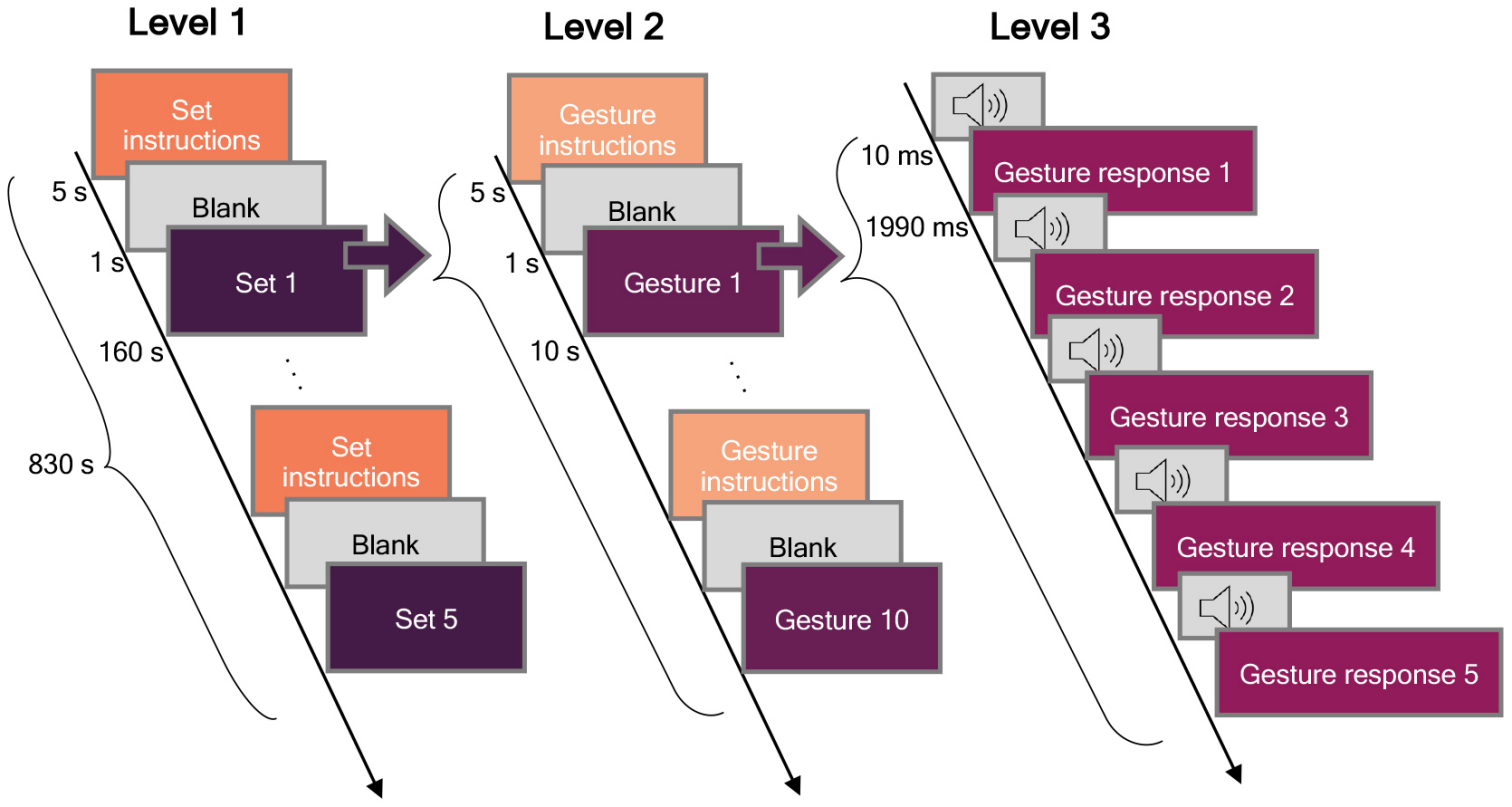
Gesture paradigm structure. The structure of the facial gesture task consists of three levels, depicted as columns in the figure. There were a total of five sets in each measurement (Level 1): Within each set, each gesture was presented in a random order (Level 2), and the same gesture was repeated five times in a row (Level 3).

#### 2.2.2 Picture naming paradigm

All subjects performed a picture naming paradigm after the facial gesture task using normed colour images of objects (Brodeur et al., 2010,^2014^). The structure of the picture naming paradigm is presented inFigure 3. The overall paradigm consisted of three experimental runs (Level 1 presents one run). Within each run, the vocal naming, silent naming, and observation tasks were repeated twice each. The order of these six task blocks was pseudorandomized using three different predetermined configurations. During vocal naming, subjects were instructed to say clearly and fluently aloud the name of the object in the picture presented to her/him. In the silent naming condition, the naming was performed in the subject’s mind without vocal utterances. In the observation condition, no response to the presented picture was required. Three of the subjects did not perform the observation task. During each task, 18–19 pictures were presented (Level 2) and named according to the task. In the vocal and silent naming tasks, each image was shown for 500 ms, after which there was a 2500 ms to respond, followed by a fixation cross for 1000 ms before the next stimulus (Level 3). In the observation task, each image was shown for 500 ms, and the observation period that followed the figure lasted only 1500 ms. In the 3 runs in total, 110 images were shown during each task.

**Fig. 3.**
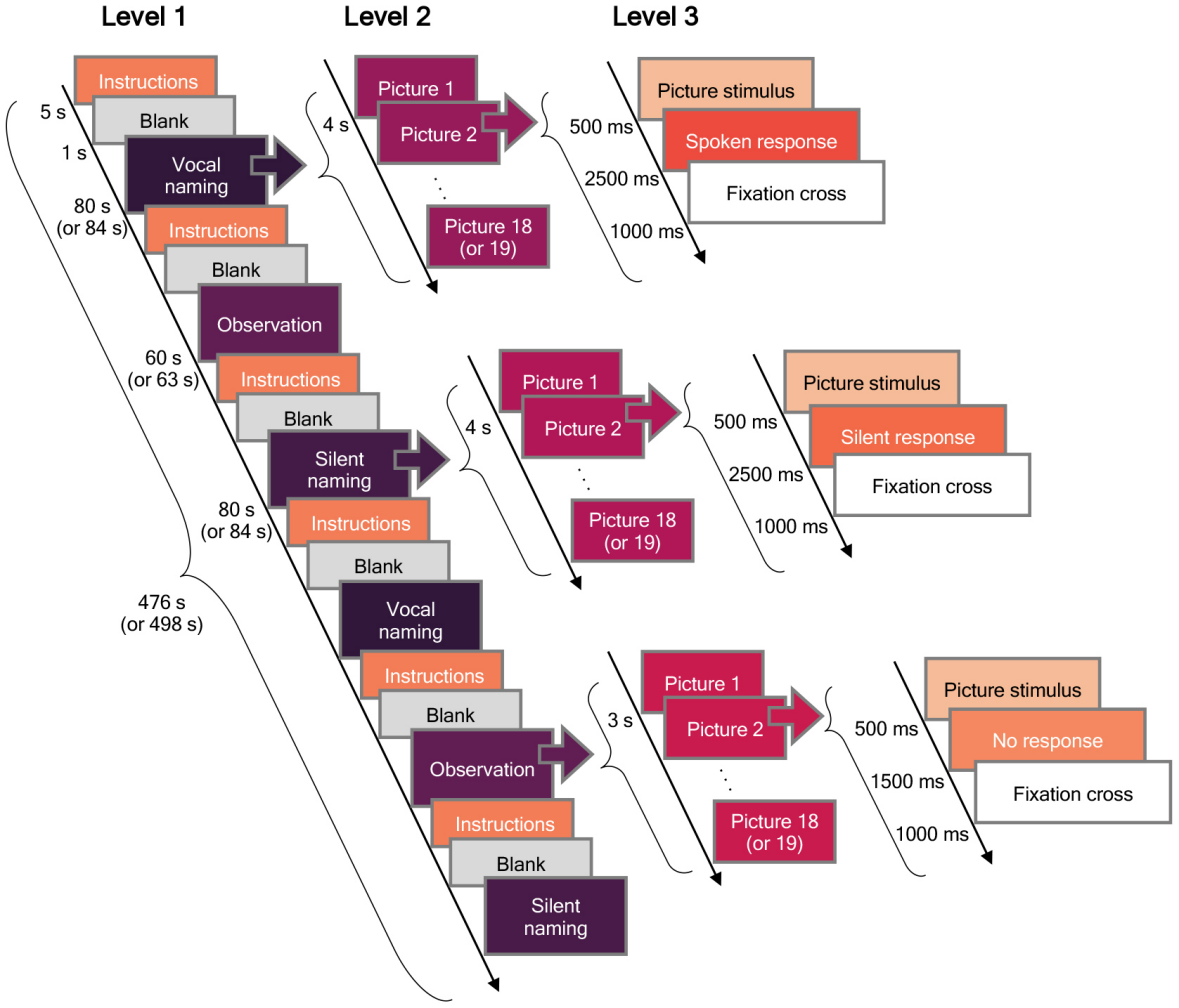
Picture naming paradigm. The paradigm consisted of three runs, that is, Level 1 was repeated three times. During each task (vocal naming, silent naming, and observation), 18–19 pictures were shown (Level 2). The number of the pictures depended on the run. Picture stimulus and response durations are shown at Level 3.

### 2.3 Recordings

The MEG recordings were conducted in the BioMag Laboratory in HUS Diagnostic Center using a 306-channel whole-head MEG device (Elektra Neuromag TRIUX, MEGIN Ltd, Helsinki, Finland) placed in a magnetically shielded room (Euroshield, Eura, Finland). The device contains 102 three-channel units, each consisting of 1 magnetometer and 2 planar gradiometers. The sampling frequency was 1000 Hz with a bandpass of 0.03–330 Hz. Five head position indicator (HPI) coils were attached to the face and scalp, and their locations were digitized with a 3-D digitizer pen (Fastrak, Polhemus, US). The 3-D digitizer was also used to digitize the subject’s head shape and three anatomical landmarks (the nasion and the right and the left preauricular points). To track the subject’s head position, the HPI coil positions were measured continuously during the whole MEG measurement by feeding a small current into them. Structural MRIs were acquired at either Aalto Magnetic Imaging Centre (Aalto University, Espoo) or at HUS with a 3T Siemens Skyra MRI scanner.

ECG and EOG were recorded alongside the EMG and MEG data. Two EOG sensor pairs for recording the horizontal and vertical eye movements were attached according toFigure 4.

**Fig. 4.**
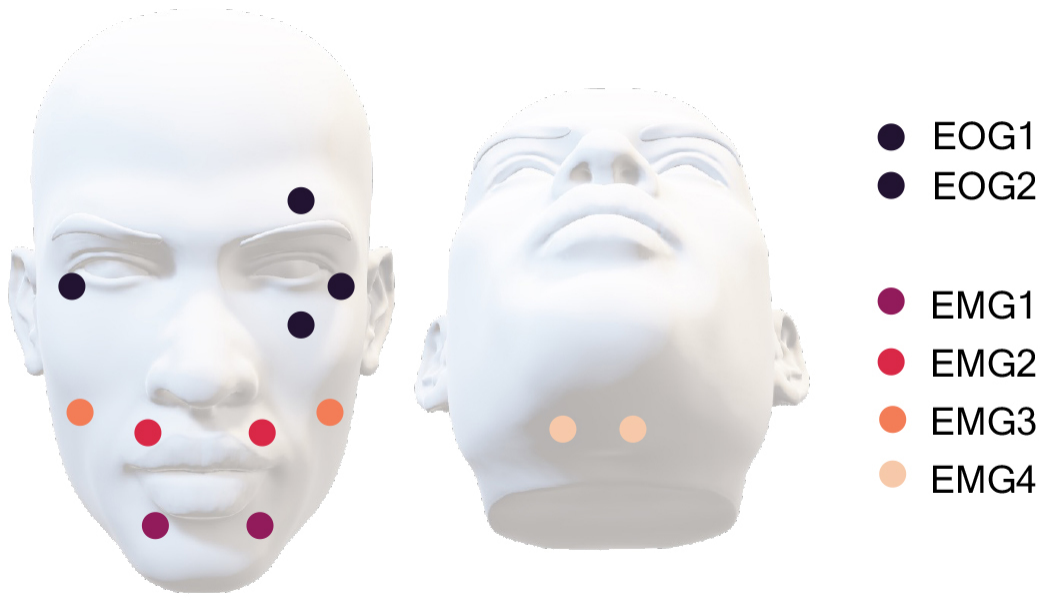
Placements of the EOG and EMG sensors. EOG1 and EOG2 represent the placements of the electrooculogram sensors that measured the horizontal and vertical eye movements. The facial muscle activity was measured with sensors EMG1–EMG4.

Muscle activation was recorded with four surface EMG sensor pairs. The positions of the sensors are visualized inFigure 4. Two EMG sensor pairs were placed above the orbicularis oris superioris (EMG1) and inferioris muscles (EMG2), respectively, similarly toAbbasi et al. (2021). The third EMG sensor pair was placed above the zygomaticus muscle, and the fourth pair under the chin, aiming to measure the muscle activity close to the rim of the MEG sensor array and activity arising from the tongue, respectively.

### 2.4 Data pre-processing

In data pre-processing, we followed standard procedures to conduct reproducible MEG data analysis (Gross et al., 2013;Jas et al., 2018). First, the temporally extended signal space separation (tSSS) algorithm (Taulu & Kajola, 2005;Taulu & Simola, 2006) was applied to the data. The method corrects head movements during the MEG measurement and removes noise emerging from sources outside the sensor array. Data from all subjects were then transformed to a common head position. Subsequently, the FastICA algorithm (Hyvärinen, 1999) implemented in MNE Python (Gramfort et al., 2013) was used to remove artefacts originating from the heart and eye movements. Only gradiometer data (204 channels) were used in the analysis. The data were then downsampled to 200 Hz. After pre-processing, the rank of the data was between 65 and 70, for all subjects.

### 2.5 Speech artefact removal

We compared three different methods for removing the speech artefact. The manual approach (Manual-ICA) provided the reference for the two automated speech artefact removal methods, MIC-ICA-N and MIC-ICA-GN. The three methods differed in terms of which data were used for the ICA decomposition, and how the artefactual independent components (ICs) were selected, as summarized inFigure 1.

#### 2.5.1 ICA decomposition

ICA (Hyvärinen, 1999,^2013^;Hyvärinen & Oja, 2000) is widely used as an artefact removal method, also for speech artefacts (Abbasi et al., 2021;Alexandrou et al., 2017;Bourguignon et al., 2020;McMenamin et al., 2010;Porcaro et al., 2015). In this work, the ICA decomposition for the speech artefact removal was conducted using the FastICA algorithm in MNE Python (Gramfort et al., 2013;Pedregosa et al., 2011). FastICA requires pre-whitening of the data. Here the pre-whitening of the input signals was conducted through principal component (PC) decomposition. The FastICA algorithm, as implemented here, therefore, requires deciding either the number of PCs or the desired amount of variance explained by the extracted components for defining the number of PCs to be retained. To avoid loss of information, we required 99.99%of the variance in the data to be explained by the PC decomposition. The ICA decomposition eventually resulted in approximately 68±3 ICA components per participant, mirroring the number of PCs extracted during the preprocessing. The artefactual components were selected from the resulting ICA components and subsequently removed. The data used to fit the ICA were approximately 1460 s for MIC-ICA-N and Manual-ICA, and 2290 s for MIC-ICA-GN, resulting in 292 200 (MIC-ICA-N and Manual-ICA) and 458 200 (MIC-ICA-GN) time samples per channel.

#### 2.5.2 Manual-ICA for a reference approach

In the reference approach, the ICA model was fit using the data from the picture naming paradigm (including the vocal naming, silent naming, and observation tasks). The naming paradigm was conducted in three runs which were subsequently concatenated, and inter-run breaks were removed. The criteria to exclude the artefact components were: (1) visual inspection of the component time series and (2) visual inspection of the component topography. SeeFigure 5for an example of a typical speech artefact IC time series and its topography. The time course resembles the simultaneously recorded EMG signal. Furthermore, the maximum foci in the topography map are located over the left and right frontotemporal channels where maximum signals from facial muscles are expected.

**Fig. 5.**
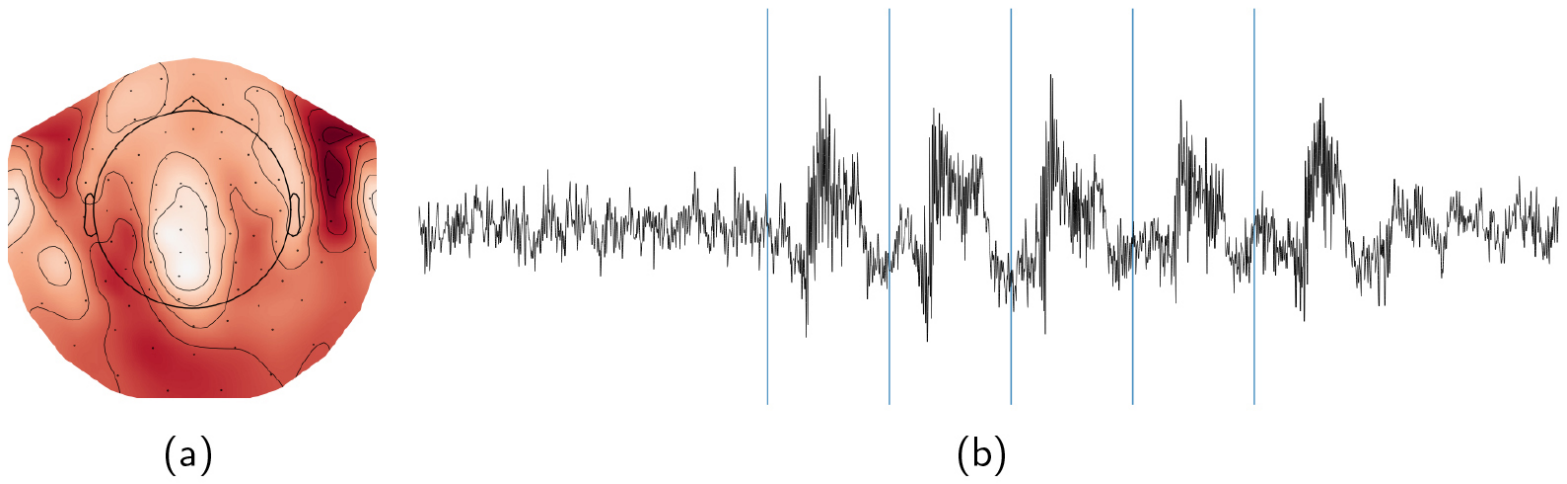
Speech artefact ICA component topography and the corresponding time series. (a) Presents the typical topography of a ICA component related to a muscle artefact, and (b) presents the time series of the component.

#### 2.5.3 MIC-ICA approaches

The MIC-ICA approaches aimed to automate the selection of the artefactual ICA components. The ICA decomposition was based on the FastICA algorithm, similar to the manual approach. The two MIC-ICA approaches utilized different data in their ICA decompositions: MIC-ICA-GN utilized both the gesture and naming paradigm data and MIC-ICA-N utilized only the naming paradigm data. For the MIC-ICA-GN, the naming paradigm data from three runs were concatenated with the gesture paradigm data. The ICA decomposition was made for the entire raw data time series, thus including the vocal naming, silent naming, and observation tasks. Hereafter the observation task data were not used in the analysis.

**Fig. 6.**
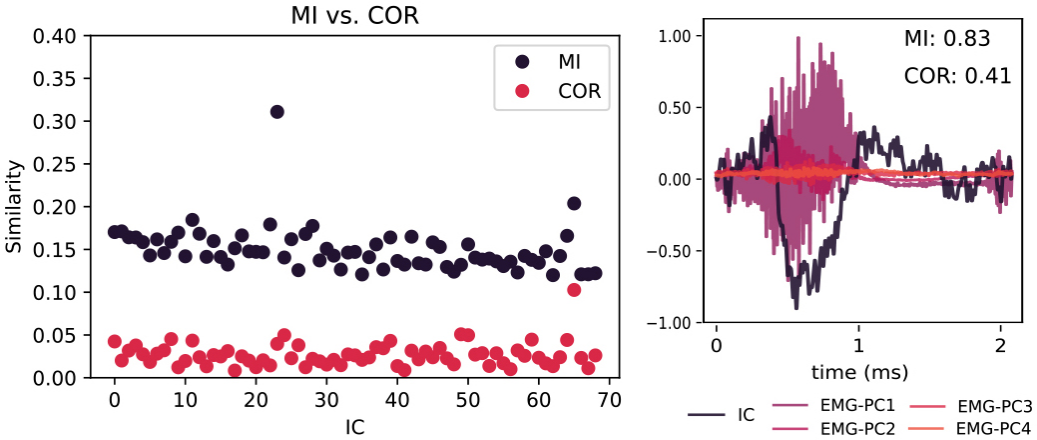
Mutual information and correlation as similarity measures. The left figure shows the mutual information and correlation calculated for one subject during the whole gesture task, demonstrating the higher potential for separating artefactual components based on mutual information than correlation. The figure on the right shows the EMG and IC signals during one gesture, justifying the reasoning for choosing MI as a similarity measure: MI can show high similarity even though the nature of the EMG and IC is not similar.

The automated component selection was based on the mutual information values between epochs of the PCA-decomposed EMG signals and the ICA-decomposed MEG sources.Figure 6shows how the mutual information and correlation between PCA decomposed EMGs and ICs behave for one subject during the whole gesture task (on the left) and during one gesture (on the right). The figure highlights the motivation for using MI instead of correlation: MI has the ability to find similarity between the EMG and the ICs, while correlation may not indicate a strong relationship between the signals being compared.

The MI values were estimated separately for the ICs of the facial gesture and vocal naming tasks and their respective EMG signals. Therefore, in the case of MIC-ICA-GN, this resulted in 8 (EMG PCA components) times∼68 (ICA components) MI values. The MIC-ICA-N approach, which utilized only the data from the vocal naming task, resulted in 4 (EMG PCA components) times∼68 (ICA components) MI values. For calculating MI, we used the Ennemi package implemented in Python (Laarne et al., 2021). Here, MI is estimated starting from its definition

$$I\left(s_{i};y_{j}\right)=\iint p\left(s_{i},y_{j}\right)\text{log}\left(\frac{p\left(s_{i},y_{j}\right)}{p\left(s_{i}\right)p\left(y_{j}\right)}\right)\text{d}s_{i}\text{d}y_{j},$$ (1)

where$p\left(s_{i},y_{j}\right)$is the joint probability distribution between the independent component$s_{i}$and the PCA-decomposed EMG component$y_{j},$and their marginal distributions are$p\left(s_{i}\right)$and$p\left(y_{j}\right)$, respectively. Mutual information takes values over[0,∞], and can be normalized to values ranging over[0,1]in different ways. Here we implemented such normalization by introducing the MI correlation coefficient (Laarne et al., 2022):

$$\rho_{I\left(s_{i};y_{j}\right)}=\sqrt{1-\text{exp}\left(-2\text{I}\left(s_{i};y_{j}\right)\right)}.$$ (2)

The Ennemi package implements a k-nearest neighbour search to estimate mutual information from continuous data (Kraskov et al., 2004;Laarne et al., 2021). Here, the parameter k was set to the library’s default value k = 3.

Finally, the components were clustered using the k-means clustering implemented in the Python Scikit-learn clustering module (Pedregosa et al., 2011) to identify artefactual components. We tested the clustering approach by varying the number of clusters from 2 to 20, finally selecting a cluster number of 5. The number of clusters was selected by visually inspecting the artefact and non-artefact clusters, searching for artefact topographies and time series similar to the Manual-ICA. We then confirmed the selectedkwith the elbow method, which calculates the sum of the squared distances between data points and their closest cluster center. The cluster number was selected such that adding more clusters no longer yielded significant improvements of the artefact identification and simultaneously avoided direct search of any single component. The independent components were ordered according to how high the MI value was for all the EMG sensors (sum of the MI values per IC). All ICs that belonged to the cluster with the highest MI sum were then selected for removal.

### 2.6 Source estimation

Source estimation was performed using MNE Python. Co-registration was performed to match the individual MRI volume and MEG sensor locations. A boundary element model (BEM) was constructed to model the conductivity profile of the head, and a source space of 2562 sources per hemisphere (ico4) was set up to define the possible current source locations. The results from the co-registration, the BEM, and the source space were used to create a forward model. A noise covariance matrix was calculated using the pre-processed epoch baselines from the silent naming task (110 trials per participant). A source estimate was obtained using noise-normalized cortically constrained minimum norm estimates (dSPM) (loose constraint = 0.3, depth weighting = 0.8). For group-level analysis, the source estimates of each subject were morphed to the Freesurfer “fsaverage” head model.

### 2.7 Evaluation of artefact removal methods

For each of the three artefact removal approaches, the vocal naming task data were reconstructed such that the artefact components obtained by each of the three methods were projected out from the data. Hereafter, we refer to this as the artefact-free data. The silent naming data were analyzed in a similar manner, by projecting out the artefact components obtained by each of the three methods, referred to as the artefact-free silent naming data. This procedure was conducted even though the artefact components were not assumed to show substantial activation during the silent naming. However, if the projected components had shown clear patterns also during silent naming, this would have indicated that the methods may not be sensitive to speech artefacts but might have removed also important brain activations. In addition, we used the original vocal naming and silent naming data without any component exclusion in the evaluation.

How well different methods preserved the brain activation was assessed by comparing the original and artefact-free evoked responses in the vocal and silent naming tasks. We assumed that there should be almost no speech-induced artefact present during the silent naming task, and thus the artefact-free and original silent naming evoked responses should not differ considerably. Furthermore, we assumed that the artefact-free and original vocal naming evoked responses would deviate from each other mainly after the speech onset. Accordingly, the less signal is (erroneously) removed before the onset of the speech, the better the brain activation is assumed to be preserved.

#### 2.7.1 Statistical testing

We tested, both at the sensor and source levels, whether the artefact-free and original vocal and silent naming data statistically differed from each other. At the sensor level, we tested all sensors independently in the time window of 0–1500 ms. We used a non-parametric cluster-level statistical permutation test (Maris & Oostenveld, 2007;Sassenhagen & Draschkow, 2019) from MNE Python version 1.3.0 (Gramfort et al., 2013) with 1000 permutations to control for multiple comparisons; the statistical significance level was set to the p-value of 0.001. The permuted epochs were individually scaled between 0 and 1, by dividing by the (individual) maximum epoch value over all channels.

The statistical significance at the source level was tested with a non-parametric cluster-level permutation test to control for multiple comparisons (Maris & Oostenveld, 2007;Sassenhagen & Draschkow, 2019) with 1000 permutations; the statistical significance level was set to a p-value of 0.05. We tested the difference between the original and cleaned vocal and silent naming task responses from the morphed source estimates, which were normalized by dividing by the maximum (individual) whole-head value. Additionally, we tested whether the cleaned vocal naming responses obtained after the different artefact removal methods differed from each other.

#### 2.7.2 Measuring the removed activation

Finally, to measure how much activation was removed in the artefact removal process, we calculated the root mean square deviation (RMSD) between the original and artefact-free evoked vocal naming data at the sensor level, independently for each speech artefact removal method. First, epochs were extracted from the vocal naming data and averaged to estimate the evoked responses. Evoked responses were windowed 0–1500 ms with respect to the image onset, aiming to include most of the spoken response into the averaged signal. Each MEG gradiometer channel pair were then combined using root mean square, after which the evoked responses were normalized by dividing them by the individual whole head maximum. The$RMSD_{ch}$values were calculated for each channel pairchindependently as follows:

$$RMSD_{ch}=\sqrt{\frac{\sum_{i=1}^{t}{\left({\hat{EV}}_{i,ch}-EV_{i,ch}\right)}^{2}}{t}},$$ (3)

where the original evoked response is marked as${\hat{EV}}_{i,ch}$, and the evoked response after artefact removal is marked as$EV_{i,ch}$. Hereiis the index of the datapoint spanning from 1 totdatapoints in the evoked response. A lower$RMSD_{ch}$value indicates greater similarity between the original and artefact-free evoked responses.

## 3 Results

### 3.1 Cortical activation during vocal and silent naming

The averaged source-level activations for the uncleaned data and for the data obtained by the different cleaning methods are depicted inFigure 7a(vocal naming) and7b(silent naming). Overall, the cleaned responses in the vocal naming task are largely similar regardless of the artefact removal method, but differ considerably from the original non-cleaned responses. The most notable differences between the original and cleaned source estimates were observed bilaterally in the anterior temporal and frontal cortices. Importantly, the source-level results for the silent naming data after cleaning were highly similar to the original ones, suggesting that the artefact removal retains the relevant language-related activations. Statistically significant differences between the cleaned and original source estimates are presented inFigure 8. AsFigure 8ashows, the brain areas that differ statistically vary slightly between the artefact removal methods: for the MIC-ICA-GN, there is no statistically significant difference in the left frontal brain areas in contrast to MIC-ICA-N and Manual-ICA. However, when testing the statistical differences between the cleaned vocal responses of different methods, none of the tested pairs (MIC-ICA-GN vs. MIC-ICA-N, MIC-ICA-GN vs. Manual-ICA, MIC-ICA-N vs. Manual-ICA) showed statistically significant differences (p<0.05). No statistical differences (p<0.05) were observed between the cleaned and original silent naming data (seeFig. 8b).

**Fig. 7.**
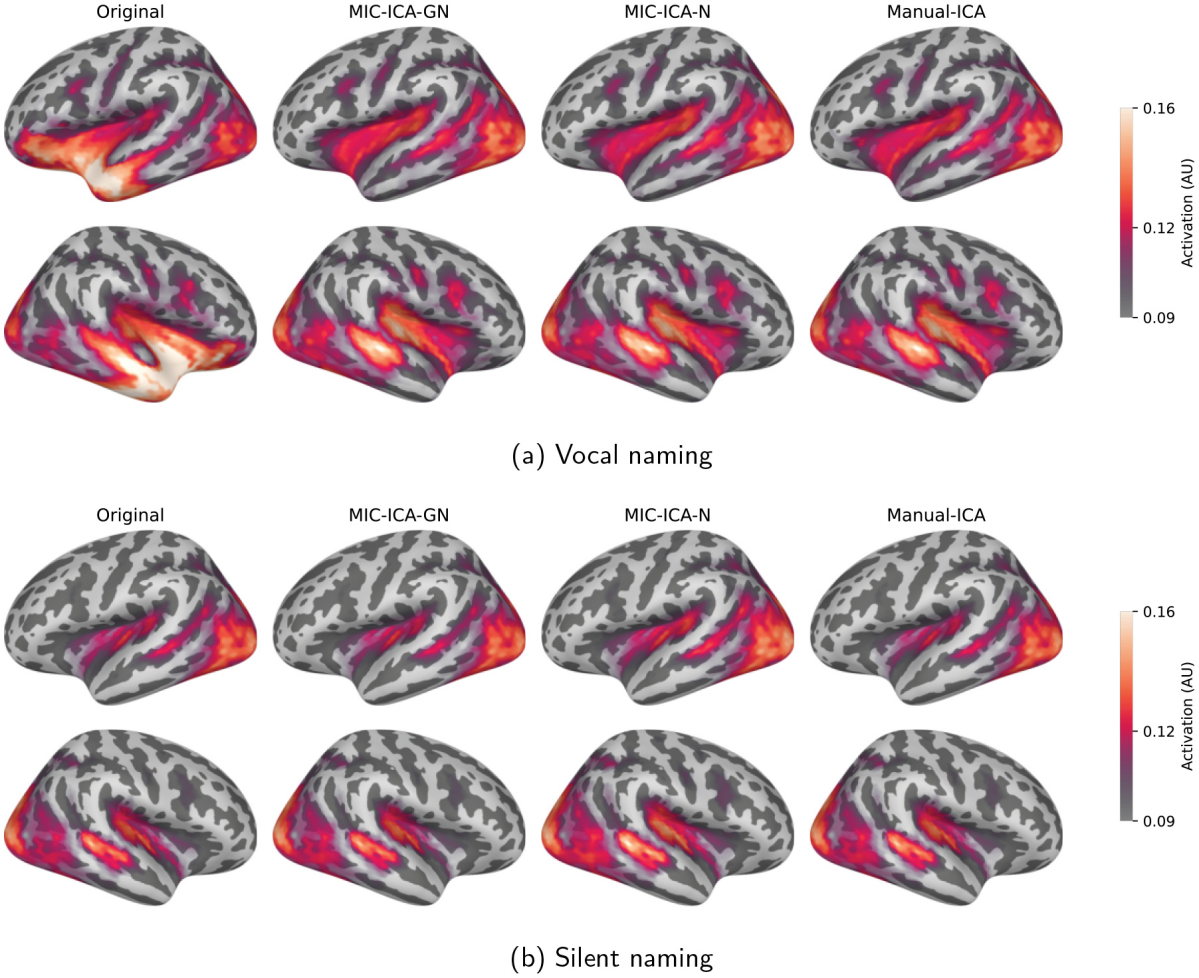
Source-level results in the vocal and silent naming tasks. Source-level results in the vocal (a) and silent naming (b) tasks, averaged over all subjects over the time interval between 0 and 1500 ms. The leftmost column shows the original brain responses and the three other columns show the cleaned activity after the three different artefact removal methods.

**Fig. 8.**
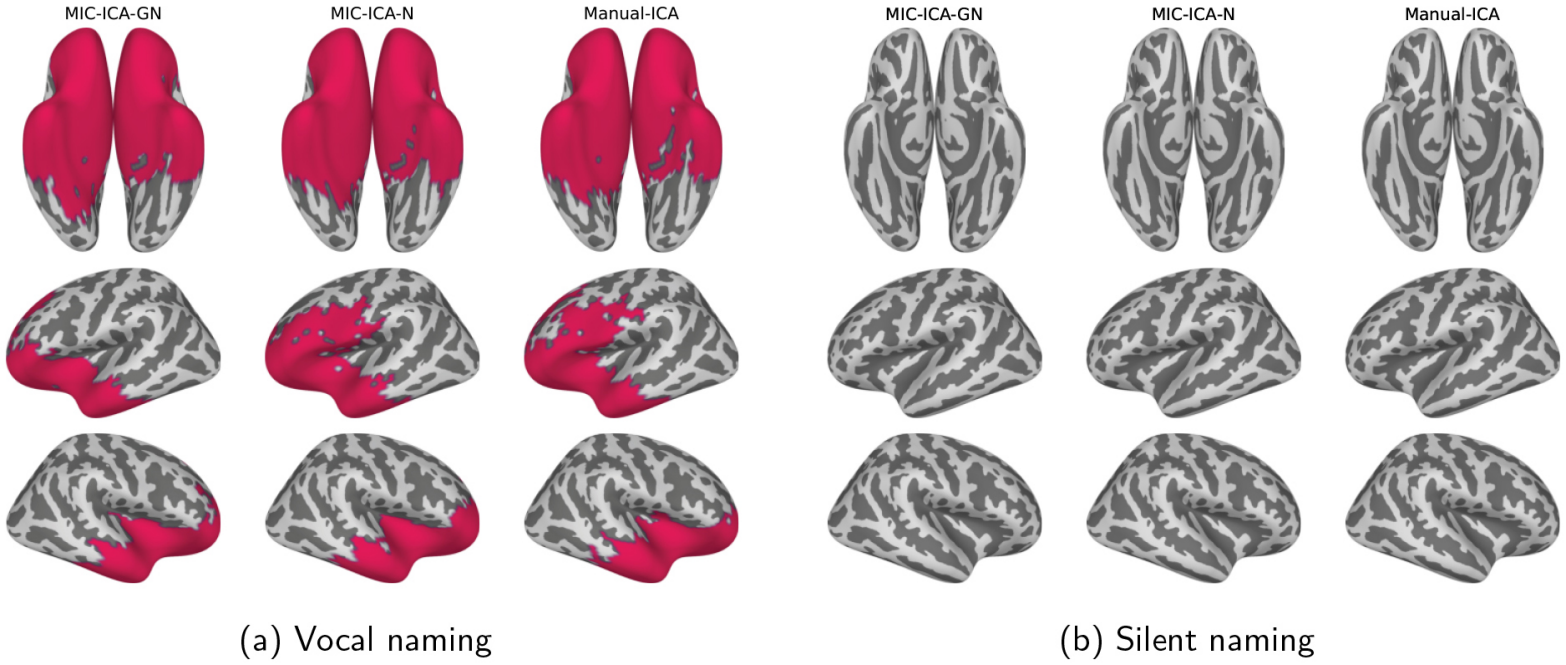
Original versus cleaned data in the vocal (a) and silent (b) naming tasks. The coloured areas visualize the source areas that differed statistically (p<0.05) between the cleaned and original data. All the areas with statistically significant differences between 0 and 1500 ms are highlighted. No statistically significant differences were observed between the cleaned and original data in the silent naming task.

Despite similarities at the group level, the differences between the artefact removal approaches became apparent at the individual level. Changing the number of clusterskwas reflected in how many components were classified as artefactual, and, therefore, assigned to be removed. The average and median number of removed components are presented inTable 2, together with a range of how many components were removed across all subjects. Typically, when a high number of components (>4) were assigned to be removed, visual inspection showed that all of these did not resemble typical speech artefact components. Therefore, the number of clusters was selected such that it would not classify too many components as artefactual and thus set tok=5, which is shown in the elbow plot inFigure 9. Based on the median, average, and min.-max. range, it seems that the MC-ICA-GN is slightly more robust, since already withk=3clusters, the MIC-ICA-GN removes a maximum of four components across all subjects and altogether the MIC-ICA-GN removes, on average, fewer components.

**Fig. 9.**
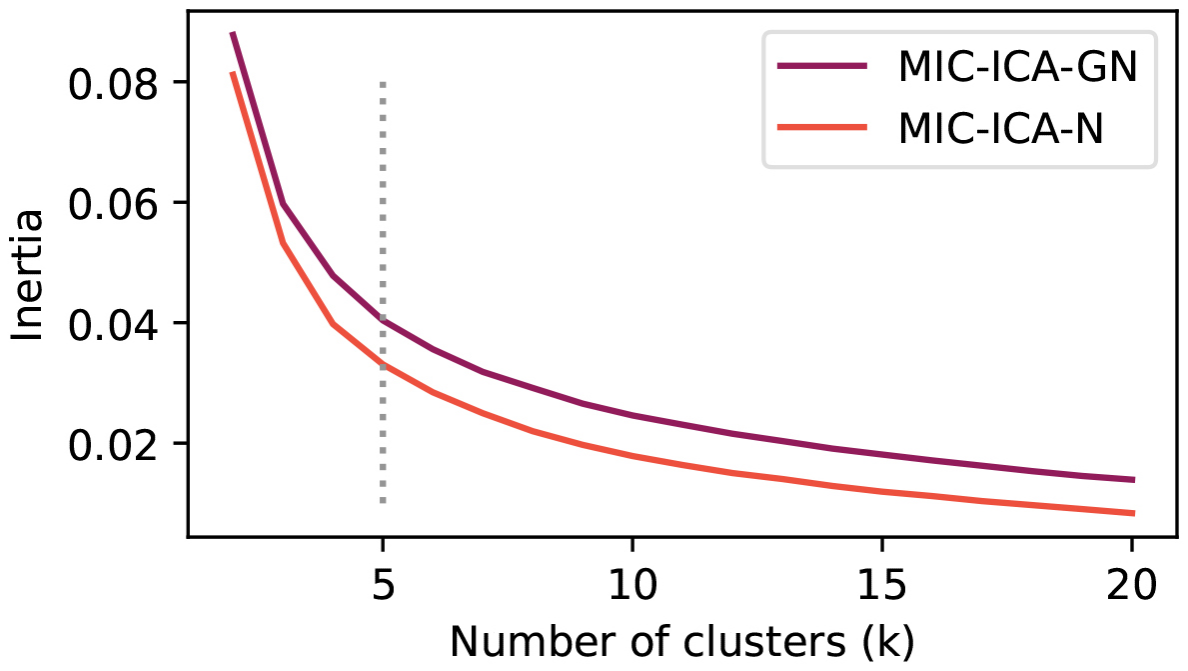
Elbow curve averaged over subjects. Inertia averaged over all subjects with different cluster sizes. The grey dotted line is drawn tok=5, which was selected to be the cluster size.

**Table 2.** Effect of the number of clusters (k) on the number of artefactually classified ICA components.

Number of clusters ( k )	2	3	4	5	6	7	8	9	10
MIC-ICA-N									
Median	5.5	3.5	1	1	1	1	1	1	1
Average	7.1	4.3	2.2	2.1	1.4	1.3	1.3	1.3	1.3
min.-max.	1-20	1-14	1-7	1-7	1-5	1-5	1-5	1-5	1-5

MIC-ICA-GN									
Median	2	1	1	1	1	1	1	1	1
Average	4.7	1.6	1.6	1.2	1.1	1.1	1.1	1.1	1.1
min.-max.	1-21	1-4	1-4	1-2	1-2	1-2	1-2	1-2	1-2

An example of individual source-level activations during vocal and silent naming is depicted inFigure 10. In this subject, the different methods removed 1, 1, and 2 components (MIC-ICA-N, MIC-ICA-GN, Manual-ICA). For the individual, the source-level activation patterns did not differ much between the different cleaning methods, while the original and cleaned data always differed clearly from each other. In contrast, the cleaning did not change the activation patterns for the silent naming data.

**Fig. 10.**
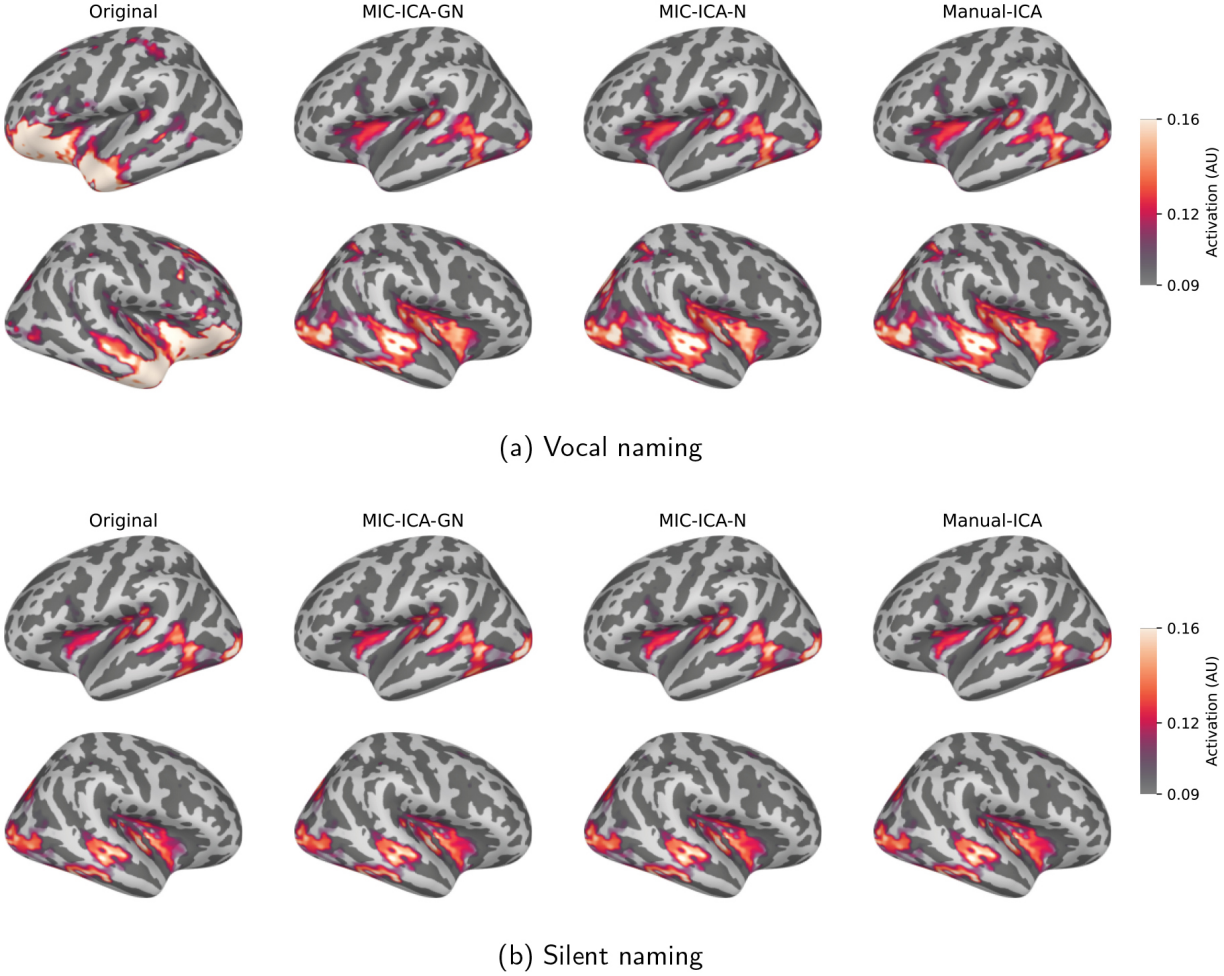
Source-level results in one individual during the vocal (a) and silent (b) naming tasks. The leftmost column shows the original responses and the three other columns show the cleaned activity after the three different artefact removal methods.

### 3.2 Comparison between different artefact removal methods

#### 3.2.1 Sensor-level results

**Fig. 11.**
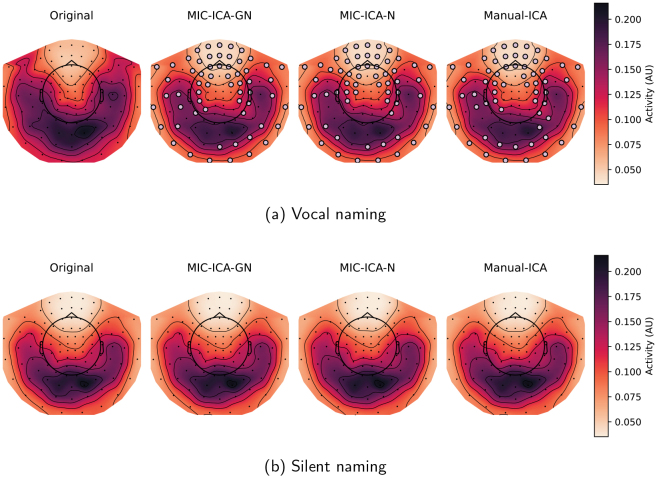
Sensor-level topographies of the vocal (a) and silent (b) naming data before and after cleaning. The data are averaged over all subjects in the time window of 0–1500 ms. The leftmost column shows the original response and the other columns show the cleaned activity during the vocal or silent naming tasks for the three different artefact removal methods. The sensor elements marked with dots mark the statistically significant differences between the cleaned versus original data.

Figure 11shows the sensor-level data averaged over all subjects during the vocal and silent naming tasks (0–1500 ms) before and after the cleaning procedures. In the vocal naming task, most of the gradiometer elements (either one or both of the sensors in the pair) differed statistically (60/61/60 elements for MIC-ICA-GN/MIC-ICA-N/Manual-ICA of which 13/11/14 were pairs) between the original and the cleaned data (p<0.001). Significant differences between the original and cleaned evoked responses were found in the time intervals of 140–1230 ms, 140–1240 ms, and 140–1240 ms for MIC-ICA-GN, MIC-ICA-N, and Manual-ICA, respectively. For the silent naming task, there were no statistically significant differences between the cleaned and original evoked responses for any of the methods.

**Fig. 12.**
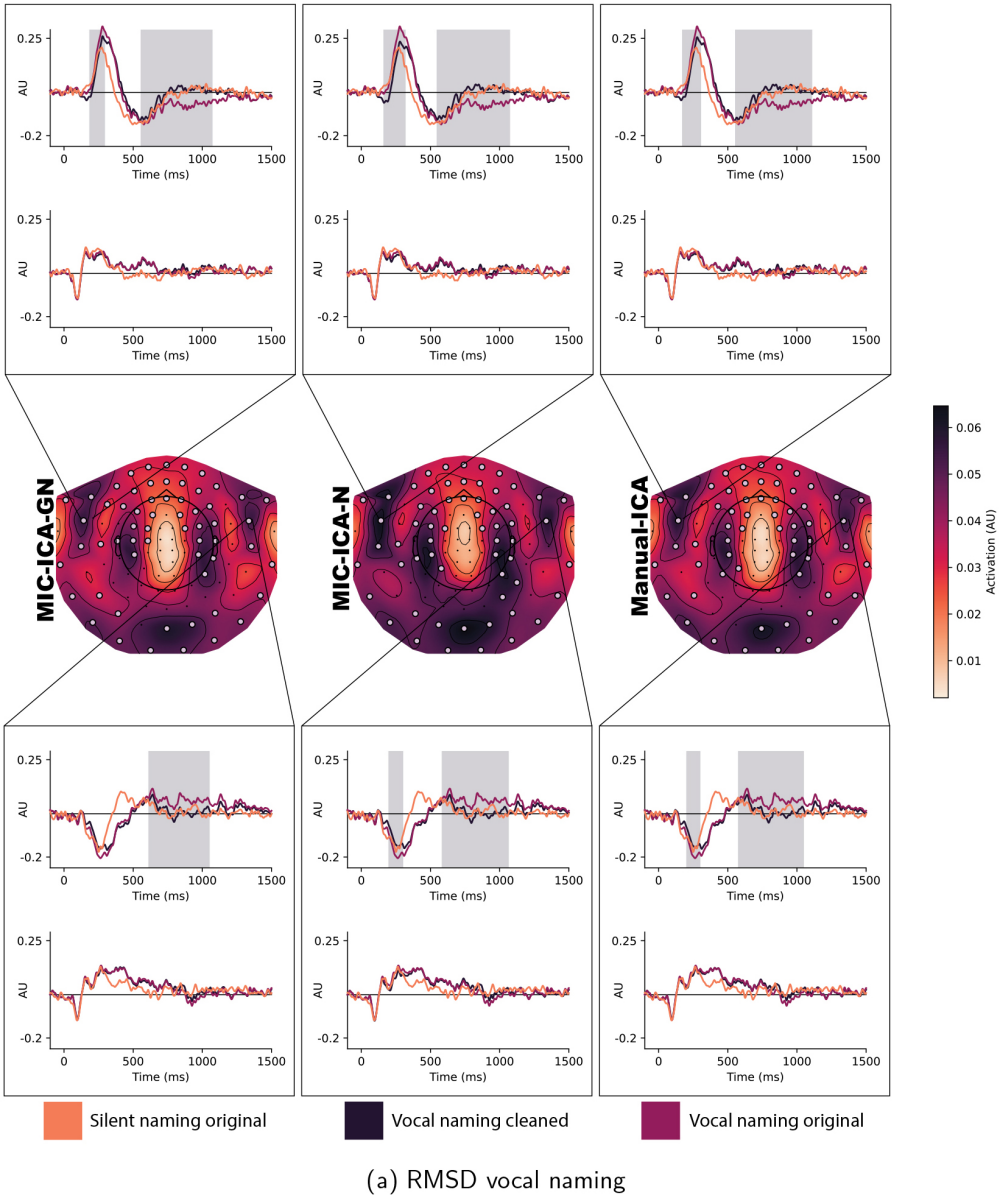

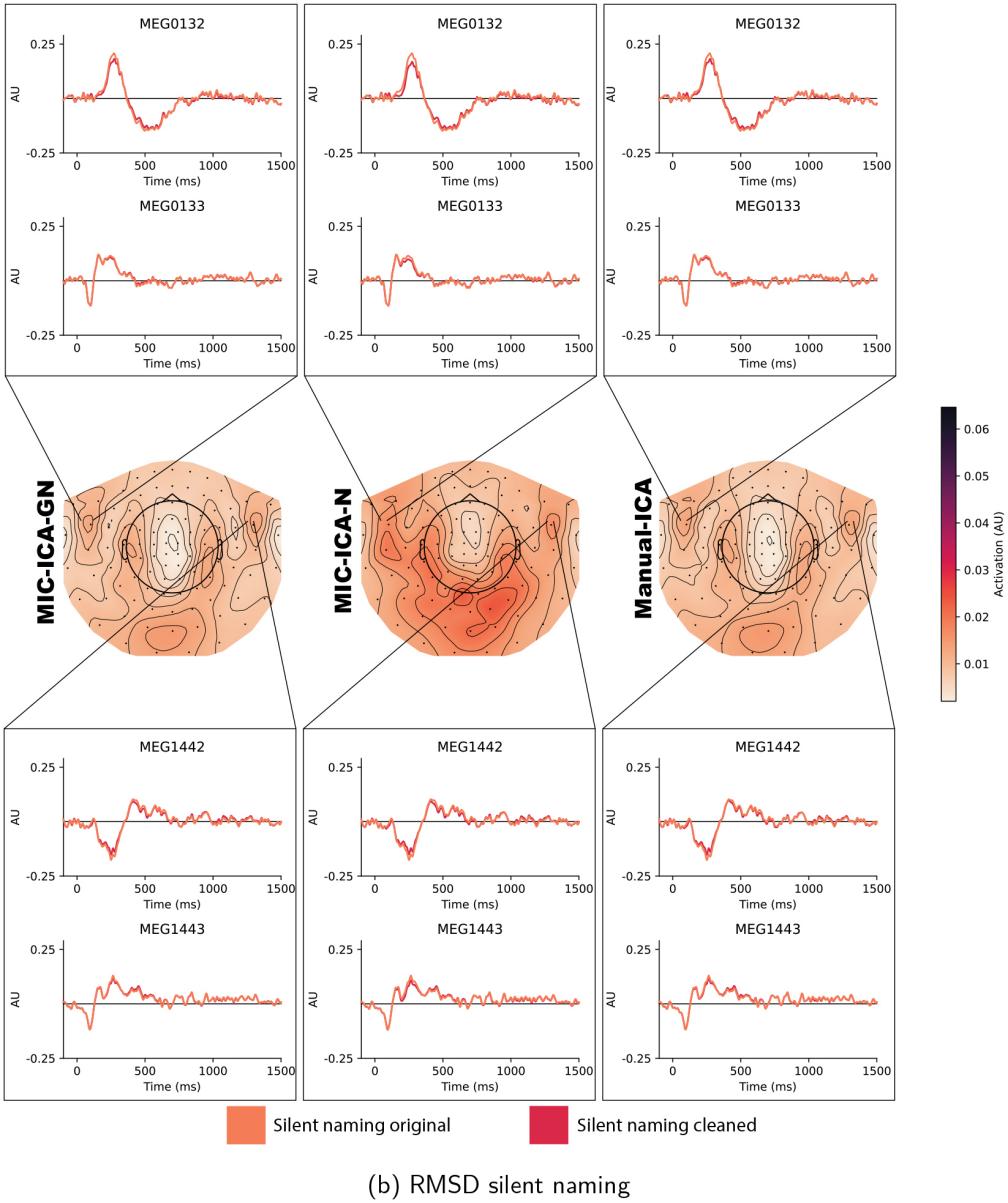
RMSD topography of the removed artefact. The middle row shows the averaged$RMSD_{ch}$values over all subjects, at each MEG sensor pair illustrating the removed amount of artefact from the vocal naming data (a) and silent naming data (b). Sensors that showed statistically significant differences between the original and cleaned data are highlighted in the topographies. The insets present the grand average evoked responses at two sensor pairs residing over areas that are relevant for language processing. The grey area in the insets shows the time during which the original and cleaned vocal conditions differed significantly.

To describe the removed signal in more detail,$RMSD_{ch}$values comparing the original and the cleaned evoked responses in the vocal and silent naming task were calculated for each subject independently and averaged into a group-level topography map (Fig. 12a, b). The obtained topography maps, describing the amount of removed artefact, were highly similar across methods. The$RMSD_{ch}$topographical maps for vocal naming have five local maxima (seeFig. 12a). Two of these maxima, one on the left side and the other on the right, reside over areas that are considered to be relevant for language processing. The evoked responses at these sensor pairs are presented in the inset below and above the topographic maps inFigure 12a and b. The shown evoked responses are grand averages over all subjects. The grey shadowed area in the insets marks the statistical difference(p<0.001)between the cleaned and original evoked responses in the vocal naming task inFigure 12aand silent naming task inFigure 12b. The cleaned vocal naming evoked response for each method seems to be similarly different to the original evoked response. Additionally, particularly around 500 ms after the stimulus onset, the cleaned vocal naming evoked responses follow closely the original silent naming responses. Despite the artefact removal, the silent naming response remains the same.

## 4 Discussion

Studying speech production with neuroimaging methods is complicated by the presence of significant artefacts in the measured brain activity during vocal speech. This study aimed to develop an ICA-based speech artefact removal routine for MEG data by (a) using EMG data measured from facial muscles during a facial gesture task for isolating the speech-induced artefacts and (b) automating the artefact component selection by utilizing mutual information (MI) and independent component (IC) approaches. Our results show that the automated MIC-ICA approaches produced highly similar results to the manual selection of artefact components. Additionally, our findings indicate that the developed facial gesture task increased the reliability of the automation. The source-level results demonstrate that the artefact removal procedure significantly changed the brain activation, especially over the anterior temporal lobe.

Previous studies have addressed the problem of speech artefacts by modifying the required speech response, for example, by using delayed (Ala-Salomäki et al., 2021;Jescheniak et al., 2002) or silent naming (Eulitz et al., 2000;Liljeström et al., 2009). Compared with these approaches, the present work used an immediate vocal response, which has significant benefits. During a vocal task, it is, for example, possible to monitor that subjects follow the task instructions correctly and identify potential speech errors. Similarly to previous solutions (Alexandrou et al., 2017;Barbati et al., 2004;Henderson et al., 2013;Porcaro et al., 2015;Vos et al., 2010), artefact removal was here based on ICA. However, the automated component selection in this study used MI between the EMG and IC signals. The use of MI was motivated by its capability to measure both linear and non-linear relationships in addition to its potential to detect high-frequency artefacts better. MI can be especially beneficial if the EMG measures are not comprehensive of all the muscle activations contributing to the artefacts, as correlation may fail in this respect. As speech artefacts are known to arise from many different sources, measuring speech artefacts more comprehensively may not be feasible: using only a few surface EMG electrodes may not be enough to address even the majority of speech artefact sources, and adding more EMGs is typically impractical.

Overall, the MIC-ICA-GN and MIC-ICA-N seemed to produce similar results to the Manual-ICA, emphasizing their benefits: reducing subjective observer bias and speeding up the artefact removal process. Neither of the automated methods clearly outperformed the other, but the MIC-ICA-GN had several advantages. The MIC-ICA-GN method affected the least the activation in the areas typically considered important to speech production, for example, the ventral frontal lobe (Levelt et al., 1998). In addition, MIC-ICA-GN affected the left and right hemispheres more symmetrically, suggesting that the ICA decomposition could better find the speech artefact with the additional facial gesture task. MIC-ICA-GN was also found to be less sensitive, in respect of the number of removed components, to the selected number of clustersk, which in turn suggests that the MIC-ICA-GN is more robust than the MIC-ICA-N. Since MIC-ICA-N could be considered to fail for a few subjects, there is uncertainty in using this method. The suggested MIC-ICA-GN approach increases the length of the recordings. For clinical applications, such as presurgical mapping of speech, it is, however, important that the method provides as accurate results at the individual level as possible and, therefore, a longer measurement time is justified. Therefore, this work promotes the use of a gesture task as a part of the study protocol, which enables reliable automation for speech artefact removal. However, the MIC-ICA-N method can still be used to speed up the removal of speech artefacts, for example, in combination with visual inspection of components. Despite these small differences, it should be emphasized that there were no visible differences in the group-level brain activation or statistical differences between the methods in pair-wise testing.

In the sensor-level analysis, the artefact reduction affected the majority of the sensors regardless of the artefact removal method. These procedures, however, did not affect the MEG signals earlier than around 140 ms, suggesting that no significant signal was removed in the early time windows. In addition, the artefact-free vocal naming and silent naming responses were largely similar to each other, especially after 500 ms. Before 500 ms, some differences were detectable, but they might also relate to the differences in the brain processes underlying vocal and silent naming. It should thus be noted that silent naming and vocal naming may also differ in terms of neural activity. It can be expected that the brain activation during vocal naming involves, for example, preparation for articulation and motor planning in addition to the vocal output (Levelt et al., 1999). Thus, the fact that the methods did not significantly remove activation from silent naming does not fully guarantee that no brain activations were removed from the vocal naming data. However, comparing these two tasks was considered the best method for evaluating the preservation of brain activation after artefact removal.

A limitation of the current study lies in the fact that all chosen methods use ICA, and there is no certainty that the ICA separates sources perfectly, such that no brain activation is mixed with the artefactual components. Furthermore, the true brain activation during speech is not known, introducing uncertainty in the evaluation of the results. Nevertheless, Manual-ICA can be taken as the current golden standard for cleaning the speech artefacts without addressing how perfectly it separates the artefact and the speech-related brain activations. Understanding this limitation, our study does not claim that MIC-ICA-GN perfectly cleans the measured MEG data from speech artefacts, but it allows to obtain results that are as good as the results from Manual-ICA in an automated way. This conclusion is supported by the results demonstrating that the artefact-free vocal naming data closely resembled the silent naming data. The size of the dataset was relatively small. However, our aim here was to extract artefact patterns which are relatively uniform across subjects, not to distinguish between individual artefact characteristics, and for this the current data set appeared demonstrative enough. However, in the future, it would be interesting to address the generalizability of our approach, for example, in children who may have different facial gesture patterns than adults. In clustering, the choice ofkis based on visual confirmation which can introduce some subjectivity to the method. Moreover, the choice ofkwas a compromise over subjects and methods. The decision could be further strengthened by automating and individualizing the cluster sizekselection in the future.

The EMG sensor placements used in this study have been previously used in M/EEG speech artefact studies (Abbasi et al., 2021;Porcaro et al., 2015), and in speech recognition studies (Meltzner et al., 2018). However, the optimal EMG placement to measure speech might not be the optimal placement for finding the artefact sources, since EMG signals that recognize speech well do not necessarily indicate high mutual information with the MEG artefact sources. Similarly to the EMG placement optimization, the gestures used here are known to produce strong muscular activation (Stepp, 2012), but whether all of them are important or needed to best imitate the speech artefact and to automate the artefact reduction was not addressed here. Future studies could address the optimal EMG placement and gesture task duration and structure. This study provides the basis for their development, as well as for a reliable evaluation of the results.

Notwithstanding these limitations, this study suggests that using the designed facial gesture task and simultaneous EMG measurements together with language-related MEG tasks improves the quality of the automated speech artefact removal process. Furthermore, the use of mutual information and clustering can help in providing an automated solution for the artefact component selection.

## Data Availability

Based on the Finnish data protection legislation, the MEG data used in this study cannot be made publicly available. The codes used for the developed methods and evaluation are made openly available athttps://github.com/BioMag/speech_artefact_removal.

## References

[b1] Abbasi , O. , Dammers , J. , Arrubla , J. , Warbrick , T. , Butz , M. , Neuner , I. , & Shah , N. J. ( 2015 ). Time-frequency analysis of resting state and evoked EEG data recorded at higher magnetic fields up to 9.4 T . Journal of Neuroscience Methods , 255 , 1 – 11 . 10.1016/j.jneumeth.2015.07.011 26213220

[b2] Abbasi , O. , Hirschmann , J. , Schmitz , G. , Schnitzler , A. , & Butz , M. ( 2016 ). Rejecting deep brain stimulation artefacts from MEG data using ICA and mutual information . Journal of Neuroscience Methods , 268 , 131 – 141 . 10.1016/j.jneumeth.2016.04.010 27090949

[b3] Abbasi , O. , Steingräber , N. , & Gross , J. ( 2021 ). Correcting meg artifacts caused by overt speech . Frontiers in Neuroscience , 15 , 682419 . 10.3389/fnins.2021.682419 34168536 PMC8217464

[b4] Ala-Salomäki , H. , Kujala , J. , Liljeström , M. , & Salmelin , R. ( 2021 ). Picture naming yields highly consistent cortical activation patterns: Test–retest reliability of magnetoencephalography recordings . NeuroImage , 227 , 117651 . 10.1016/j.neuroimage.2020.117651 33338614

[b5] Alexandrou , A. M. , Saarinen , T. , Mäkelä , S. , Kujala , J. , & Salmelin , R. ( 2017 ). The right hemisphere is highlighted in connected natural speech production and perception . NeuroImage , 152 , 628 – 638 . 10.1016/j.neuroimage.2017.03.006 28268122

[b6] Aristei , S. , Melinger , A. , & Rahman , R. A. ( 2011 ). Electrophysiological chronometry of semantic context effects in language production . Journal of Cognitive Neuroscience , 23 ( 7 ), 1567 – 1586 . 10.1162/jocn.2010.21474 20515409

[b7] Barbati , G. , Porcaro , C. , Zappasodi , F. , Rossini , P. M. , & Tecchio , F. ( 2004 ). Optimization of an independent component analysis approach for artifact identification and removal in magnetoencephalographic signals . Clinical Neurophysiology , 115 ( 5 ), 1220 – 1232 . 10.1016/j.clinph.2003.12.015 15066548

[b8] Bourguignon , M. , Molinaro , N. , Lizarazu , M. , Taulu , S. , Jousmäki , V. , Lallier , M. , Carreiras , M. , & De Tiege , X . ( 2020 ). Neocortical activity tracks the hierarchical linguistic structures of self-produced speech during reading aloud . NeuroImage , 216 , 116788 . 10.1016/j.neuroimage.2020.116788 32348908

[b9] Brodeur , M. B. , Dionne-Dostie , E. , Montreuil , T. , & Lepage , M. ( 2010 ). The bank of standardized stimuli (boss), a new set of 480 normative photos of objects to be used as visual stimuli in cognitive research . PLoS One , 5 ( 5 ), e10773 . 10.1371/journal.pone.0010773 20532245 PMC2879426

[b10] Brodeur , M. B. , Guérard , K. , & Bouras , M. ( 2014 ). Bank of standardized stimuli (BOSS) phase II: 930 new normative photos . PLoS One , 9 ( 9 ), e106953 . 10.1371/journal.pone.0106953 25211489 PMC4161371

[b11] Corina , D. P. , Loudermilk , B. C. , Detwiler , L. , Martin , R. F. , Brinkley , J. F. , & Ojemann , G. ( 2010 ). Analysis of naming errors during cortical stimulation mapping: Implications for models of language representation . Brain and Language , 115 ( 2 ), 101 – 112 . 10.1016/j.bandl.2010.04.001 20452661 PMC3247200

[b12] Correa , C. D. , & Lindstrom , P. ( 2013 ). The mutual information diagram for uncertainty visualization . International Journal for Uncertainty Quantification , 3 ( 3 ), 187 – 201 . 10.1615/int.j.uncertaintyquantification.2012003959

[b13] Eulitz , C. , Hauk , O. , & Cohen , R. ( 2000 ). Electroencephalographic activity over temporal brain areas during phonological encoding in picture naming . Clinical Neurophysiology , 111 ( 11 ), 2088 – 2097 . 10.1016/s1388-2457(00)00441-7 11068246

[b14] Fargier , R. , Buerki , A. , Pinet , S. , Alario , F.-X. , & Laganaro , M. ( 2018 ). Word onset phonetic properties and motor artifacts in speech production EEG recordings . Psychophysiology , 55 ( 2 ), e12982 . 10.1111/psyp.12982 28850684

[b15] Ganushchak , L. Y. , & Schiller , N. O. ( 2008 ). Motivation and semantic context affect brain error-monitoring activity: An event-related brain potentials study . Neuroimage , 39 ( 1 ), 395 – 405 . 10.1016/j.neuroimage.2007.09.001 17920932

[b16] Gramfort , A. , Luessi , M. , Larson , E. , Engemann , D. A. , Strohmeier , D. , Brodbeck , C. , Goj , R. , Jas , M. , Brooks , T. , Parkkonen , L. , & Hämäläinen , M. S. ( 2013 ). MEG and EEG data analysis with MNE-Python . Frontiers in Neuroscience , 7 ( 267 ), 1 – 13 . 10.3389/fnins.2013.00267 24431986 PMC3872725

[b17] Gross , J. , Baillet , S. , Barnes , G. R. , Henson , R. N. , Hillebrand , A. , Jensen , O. , Jerbi , K. , Litvak , V. , Maess , B. , Oostenveld , R. , Parkkonen , L. , Taylor , J. R. , van Wassenhove , V. , Wibral , M. , & Schoffelen , J.-M. ( 2013 ). Good practice for conducting and reporting meg research . NeuroImage , 65 , 349 – 363 . 10.1016/j.neuroimage.2012.10.001 23046981 PMC3925794

[b18] Habets , B. , Jansma , B. M. , & Münte , T. F. ( 2008 ). Neurophysiological correlates of linearization in language production . BMC Neuroscience , 9 ( 1 ), 1 – 8 . 10.1186/1471-2202-9-77 18681961 PMC2543022

[b19] Henderson , J. M. , Luke , S. G. , Schmidt , J. , & Richards , J. E. ( 2013 ). Co-registration of eye movements and event-related potentials in connected-text paragraph reading . Frontiers in Systems Neuroscience , 7 , 28 . 10.3389/fnsys.2013.00028 23847477 PMC3706749

[b20] Hipp , J. F. , Engel , A. K. , & Siegel , M. ( 2011 ). Oscillatory synchronization in large-scale cortical networks predicts perception . Neuron , 69 ( 2 ), 387 – 396 . 10.1016/j.neuron.2010.12.027 21262474

[b21] Hudson , J. E. ( 2006 ). Signal processing using mutual information . IEEE Signal Processing Magazine , 23 ( 6 ), 50 – 54 . 10.1109/sp-m.2006.248712

[b22] Hyvärinen , A. ( 1999 ). Fast and robust fixed-point algorithms for independent component analysis . IEEE Transactions on Neural Networks , 10 ( 3 ), 626 – 634 . 10.1109/72.761722 18252563

[b23] Hyvärinen , A. ( 2013 ). Independent component analysis: Recent advances . Philosophical Transactions of the Royal Society A: Mathematical, Physical and Engineering Sciences , 371 ( 1984 ), 20110534 . 10.1098/rsta.2011.0534 PMC3538438 23277597

[b24] Hyvärinen , A. , & Oja , E. ( 2000 ). Independent component analysis: Algorithms and applications . Neural Networks , 13 ( 4–5 ), 411 – 430 . 10.1016/s0893-6080(00)00026-5 10946390

[b25] Indefrey , P. , & Levelt , W. J. ( 2004 ). The spatial and temporal signatures of word production components . Cognition , 92 ( 1–2 ), 101 – 144 . 10.1016/j.cognition.2002.06.001 15037128

[b26] Jas , M. , Larson , E. , Engemann , D. A. , Leppäkangas , J. , Taulu , S. , Hämäläinen , M. , & Gramfort , A. ( 2018 ). A reproducible MEG/EEG group study with the MNE software: Recommendations, quality assessments, and good practices . Frontiers in Neuroscience , 12 , 530 . 10.3389/fnins.2018.00530 30127712 PMC6088222

[b27] Jescheniak , J. D. , Schriefers , H. , Garrett , M. F. , & Friederici , A. D. ( 2002 ). Exploring the activation of semantic and phonological codes during speech planning with event-related brain potentials . Journal of Cognitive Neuroscience , 14 ( 6 ), 951 – 964 . 10.1162/089892902760191162 12191461

[b28] Jiang , X. , Bian , G.-B. , & Tian , Z. ( 2019 ). Removal of artifacts from EEG signals: A review . Sensors , 19 ( 5 ), 987 . 10.3390/s19050987 30813520 PMC6427454

[b29] Kohn , S. E. , & Goodglass , H. ( 1985 ). Picture-naming in aphasia . Brain and Language , 24 ( 2 ), 266 – 283 . 10.1016/0093-934x(85)90135-x 3978406

[b30] Kraskov , A. , Stögbauer , H. , & Grassberger , P. ( 2004 ). Estimating mutual information . Physical Review E—Statistical, Nonlinear, and Soft Matter Physics , 69 ( 6 ), 066138 . 10.1103/physreve.83.019903 15244698

[b31] Krieg , S. M. , Lioumis , P. , Mäkelä , J. P. , Wilenius , J. , Karhu , J. , Hannula , H. , Savolainen , P. , Lucas , C. W. , Seidel , K. , Laakso , A. , Islam , M. , Vaalto , S. , Lehtinen , H. , Vitikainen , A.-M. , Tarapore , P. E. , & Picht , T. ( 2017 ). Protocol for motor and language mapping by navigated TMS in patients and healthy volunteers; workshop report . Acta Neurochirurgica , 159 , 1187 – 1195 . 10.1007/s00701-017-3187-z 28456870

[b32] Kujala , M. V. , Törnqvist , H. , Somppi , S. , Hänninen , L. , Krause , C. M. , Vainio , O. , & Kujala , J. ( 2013 ). Reactivity of dogs’ brain oscillations to visual stimuli measured with non-invasive electroencephalography . PLoS One , 8 ( 5 ), e61818 . 10.1371/journal.pone.0061818 23650504 PMC3641087

[b33] Laarne , P. , Amnell , E. , Zaidan , M. A. , Mikkonen , S. , & Nieminen , T. ( 2022 ). Exploring non-linear dependencies in atmospheric data with mutual information . Atmosphere , 13 ( 7 ), 1046 . 10.3390/atmos13071046

[b34] Laarne , P. , Zaidan , M. A. , & Nieminen , T. ( 2021 ). Ennemi: Non-linear correlation detection with mutual information . SoftwareX , 14 , 100686 . 10.1016/j.softx.2021.100686

[b35] Laganaro , M. , & Perret , C. ( 2011 ). Comparing electrophysiological correlates of word production in immediate and delayed naming through the analysis of word age of acquisition effects . Brain Topography , 24 ( 1 ), 19 – 29 . 10.1007/s10548-010-0162-x 20938730

[b36] Levelt , W. J. , Praamstra , P. , Meyer , A. S. , Helenius , P. , & Salmelin , R. ( 1998 ). An MEG study of picture naming . Journal of Cognitive Neuroscience , 10 ( 5 ), 553 – 567 . 10.1162/089892998562960 9802989

[b37] Levelt , W. J. , Roelofs , A. , & Meyer , A. S. ( 1999 ). A theory of lexical access in speech production . Behavioral and Brain Sciences , 22 ( 1 ), 1 – 38 . 10.1017/s0140525x99001776 11301520

[b38] Liljeström , M. , Hulten , A. , Parkkonen , L. , & Salmelin , R. ( 2009 ). Comparing MEG and fMRI views to naming actions and objects . Human Brain Mapping , 30 ( 6 ), 1845 – 1856 . 10.1002/hbm.20785 19378277 PMC6870641

[b39] Liljeström , M. , Kujala , J. , Stevenson , C. , & Salmelin , R. ( 2015 ). Dynamic reconfiguration of the language network preceding onset of speech in picture naming . Human Brain Mapping , 36 ( 3 ), 1202 – 1216 . 10.1002/hbm.22697 25413681 PMC4365727

[b40] Lioumis , P. , Zhdanov , A. , Mäkelä , N. , Lehtinen , H. , Wilenius , J. , Neuvonen , T. , Hannula , H. , Deletis , V. , Picht , T. , & Mäkelä , J. P. ( 2012 ). A novel approach for documenting naming errors induced by navigated transcranial magnetic stimulation . Journal of Neuroscience Methods , 204 ( 2 ), 349 – 354 . 10.1016/j.jneumeth.2011.11.003 22108143

[b41] Liu , Z. , de Zwart , J. A. , van Gelderen , P. , Kuo , L.-W. , & Duyn , J. H. ( 2012 ). Statistical feature extraction for artifact removal from concurrent fMRI-EEG recordings . NeuroImage , 59 ( 3 ), 2073 – 2087 . 10.1016/j.neuroimage.2011.10.042 22036675 PMC3254729

[b42] Maris , E. , & Oostenveld , R. ( 2007 ). Nonparametric statistical testing of EEG-and MEG-data . Journal of Neuroscience Methods , 164 ( 1 ), 177 – 190 . 10.1016/j.jneumeth.2007.03.024 17517438

[b43] McMenamin , B. W. , Shackman , A. J. , Maxwell , J. S. , Bachhuber , D. R. , Koppenhaver , A. M. , Greischar , L. L. , & Davidson , R. J. ( 2010 ). Validation of ICA-based myogenic artifact correction for scalp and source-localized EEG . NeuroImage , 49 ( 3 ), 2416 – 2432 . 10.1016/j.neuroimage.2009.10.010 19833218 PMC2818255

[b44] Meltzner , G. S. , Heaton , J. T. , Deng , Y. , De Luca , G. , Roy , S. H. , & Kline , J. C. ( 2018 ). Development of SEMG sensors and algorithms for silent speech recognition . Journal of Neural Engineering , 15 ( 4 ), 046031 . 10.1088/1741-2552/aac965 29855428 PMC6168082

[b45] Muthukumaraswamy , S. D. ( 2013 ). High-frequency brain activity and muscle artifacts in MEG/EEG: A review and recommendations . Frontiers in Human Neuroscience , 7 , 138 . 10.3389/fnhum.2013.00138 23596409 PMC3625857

[b46] O’Dwyer , N. J. , Quinn , P. T. , Guitar , B. E. , Andrews , G. , & Neilson , P. D. ( 1981 ). Procedures for verification of electrode placement in EMG studies of orofacial and mandibular muscles . Journal of Speech, Language, and Hearing Research , 24 ( 2 ), 273 – 288 . 10.1044/jshr.2402.273 7265944

[b47] Ouyang , G. , Sommer , W. , Zhou , C. , Aristei , S. , Pinkpank , T. , & Abdel Rahman , R . ( 2016 ). Articulation artifacts during overt language production in event-related brain potentials: Description and correction . Brain Topography , 29 , 791 – 813 . 10.1007/s10548-016-0515-1 27509898

[b48] Pedregosa , F. , Varoquaux , G. , Gramfort , A. , Michel , V. , Thirion , B. , Grisel , O. , Blondel , M. , Prettenhofer , P. , Weiss , R. , Dubourg , V. , Vanderplas , J. , Passos , A. , Cournapeau , D. , Brucher , M. , Perrot , M. , & Duchesnay , E. ( 2011 ). Scikit-learn: Machine learning in Python . Journal of Machine Learning Research , 12 , 2825 – 2830 . 10.3389/fninf.2014.00014

[b49] Porcaro , C. , Medaglia , M. T. , & Krott , A. ( 2015 ). Removing speech artifacts from electroencephalographic recordings during overt picture naming . NeuroImage , 105 , 171 – 180 . 10.1016/j.neuroimage.2014.10.049 25450111

[b50] Raffa , G. , Marzano , G. , Curcio , A. , Espahbodinea , S. , Germanò , A. , & Angileri , F. F. ( 2022 ). Personalized surgery of brain tumors in language areas: The role of preoperative brain mapping in patients not eligible for awake surgery . Neurosurgical Focus , 53 ( 6 ), E3 . 10.3171/2022.9.focus22415 39264003

[b51] Salmelin , R. , Hari , R. , Lounasmaa , O. , & Sams , M. ( 1994 ). Dynamics of brain activation during picture naming . Nature , 368 ( 6470 ), 463 – 465 . 10.1038/368463a0 8133893

[b52] Sassenhagen , J. , & Draschkow , D. ( 2019 ). Cluster-based permutation tests of MEG/EEG data do not establish significance of effect latency or location . Psychophysiology , 56 ( 6 ), e13335 . 10.1111/psyp.13335 30657176

[b53] Schmitt , B. M. , Münte , T. F. , & Kutas , M. ( 2000 ). Electrophysiological estimates of the time course of semantic and phonological encoding during implicit picture naming . Psychophysiology , 37 ( 4 ), 473 – 484 . 10.1017/s0048577200981782 10934906

[b54] Sonoda , M. , Rothermel , R. , Carlson , A. , Jeong , J.-W. , Lee , M.-H. , Hayashi , T. , Luat , A. F. , Sood , S. , & Asano , E. ( 2022 ). Naming-related spectral responses predict neuropsychological outcome after epilepsy surgery . Brain , 145 ( 2 ), 517 – 530 . 10.1093/brain/awab318 35313351 PMC9014727

[b55] Stepp , C. E. ( 2012 ). Surface electromyography for speech and swallowing systems: measurement, analysis, and interpretation . Journal of Speech, Language, and Hearing Research , 55 ( 4 ), 1232 – 1246 . 10.1044/1092-4388(2011/11-0214) 22232412

[b56] Taulu , S. , & Kajola , M. ( 2005 ). Presentation of electromagnetic multichannel data: The signal space separation method . Journal of Applied Physics , 97 ( 12 ), 124905 . 10.1063/1.1935742

[b57] Taulu , S. , & Simola , J. ( 2006 ). Spatiotemporal signal space separation method for rejecting nearby interference in meg measurements . Physics in Medicine & Biology , 51 ( 7 ), 1759 . 10.1088/0031-9155/51/7/008 16552102

[b58] Urigüen , J. A. , & Garcia-Zapirain , B. ( 2015 ). EEG artifact removal—State-of-the-art and guidelines . Journal of Neural Engineering , 12 ( 3 ), 031001 . 10.1088/1741-2560/12/3/031001 25834104

[b59] Vanhatalo , S. , Voipio , J. , Dewaraja , A. , Holmes , M. D. , & Miller , J. W. ( 2003 ). Topography and elimination of slow EEG responses related to tongue movements . NeuroImage , 20 ( 2 ), 1419 – 1423 . 10.1016/s1053-8119(03)00392-6 14568511

[b60] Vos , D. M. , Riès , S. , Vanderperren , K. , Vanrumste , B. , Alario , F.-X. , Huffel , V. S. , & Burle , B. ( 2010 ). Removal of muscle artifacts from EEG recordings of spoken language production . Neuroinformatics , 8 , 135 – 150 . 10.1007/s12021-010-9071-0 20480401

